# Sowing Wild Oats

**Published:** 1914-03

**Authors:** 


### Sowing Wild Oats.

"Wild oats" covers a multitude of sins. This is a convenient term that is used frequently by a young man's friends to excuse his rakish and dissolute conduct, and to whitewash the rottenness that comes to the surface from time to time. A young man who is in the process of sowing his "wild oats" is operating under a license that has been issued to him by common consent, and apparently there is no vice or sin anywhere between hell and breakfast that he is not at liberty to commit and go unwhipt of justice. Sowing wild oats is like the whooping cough, the measles and the mumps—there is no way to avoid them, and the sooner they are over, the sooner will the young Lothario settle down to make love to some innocent, lovable girl and persuade her to become a partner in the harvesting of his crop of wild oats.

The innocent, lovable girl is told that the young man must sow his wild oats, and that he is not qualified either for matrimony or for fatherhood until he has gone through a season of infamous immorality and shame. And in most instances she believes it all. She believes it, until some sad day she finds herself being hacked and carved by the surgeon's knife, or sees her children suffering from afflictions that give the lie to the stories that she has been told. She believes it until the harvest time comes, and until the crop of wild oats is gathered into her cradle and stored around her family fireside. She is awakened then from her girlhood dreams, but she is already in the harvest field and the duties of the day will hold her until the day is done. She discovers that her home is different only in name from a pesthouse, and that her lover was likewise a lazar and a leper.

This theory about the sowing of wild oats should have long since been exposed and exploded. In the first place it was thought out and clothed with a certificate of authority by man himself. It originated in the same brain that first preached polygamy and that placed woman in the category of chattels and dogs and human slaves. And in order that man might have a complete monopoly of wild oats sowing, a double standard of morality was fixed and woman was prohibited from becoming a sower. She was invited in only at harvest time. She might become a reaper, but man alone might sow.

Wild oats sowing is as inexcusable as is drunkenness, as is theft, as is any crime that man commits. And wild oats sowing is governed by the same inexorable law of nature that causes the acorn to produce the oak and the grain to produce its kind. It was a mandate from the Almighty, that what we sow, that shall we also reap. Man cannot change this law if he would. He cannot avoid its penalty, except by refraining from its violation. And wild oats sowing not only bears fruit in the first generation, but also in the second and in the third, and on and on until the lineal tree dies from sheer exhaustion. Nature pities the weak and permits death to bring the struggle for existence to an end.

---

The absence of displacement in a fracture of the olecranon gives no promise of immunity from impaired function; callous formation may interfere with the joint action.—*American Journal of Surgery.*

---

DURING THE CIVIL WAR the late Colonel Gabe Bouch organized a regiment which he controlled as a dictator.

"I am an humble servant of the Lord," said an itinerant evangelist who had wandered into the camp one day, "endeavoring to save the soul of the unfortunate. I have just left the camp of the —th Massachusetts, where I was instrumental in leading eight men into the paths of righteousness."

"Adjutant," thundered Colonel Bouch, after a moment's pause, "detail ten men for baptism. No d—d Massachusetts regiment shall beat mine for piety."—*The Vagabond.*

